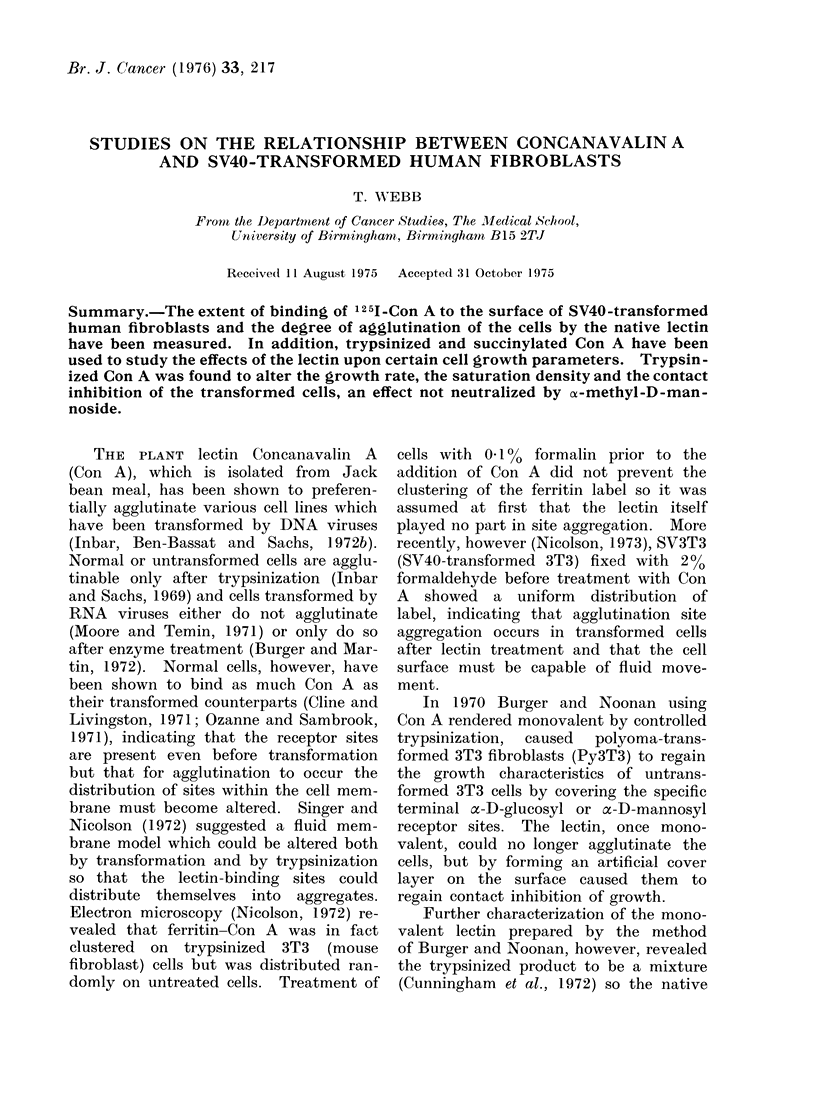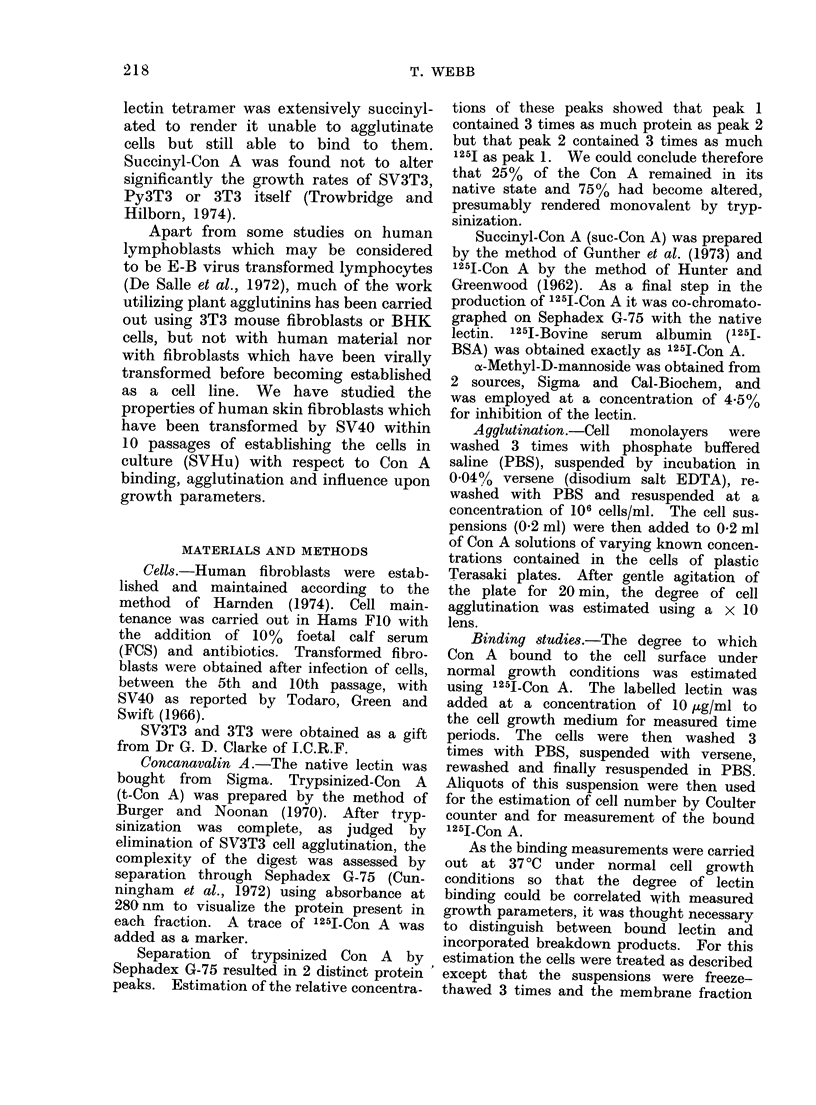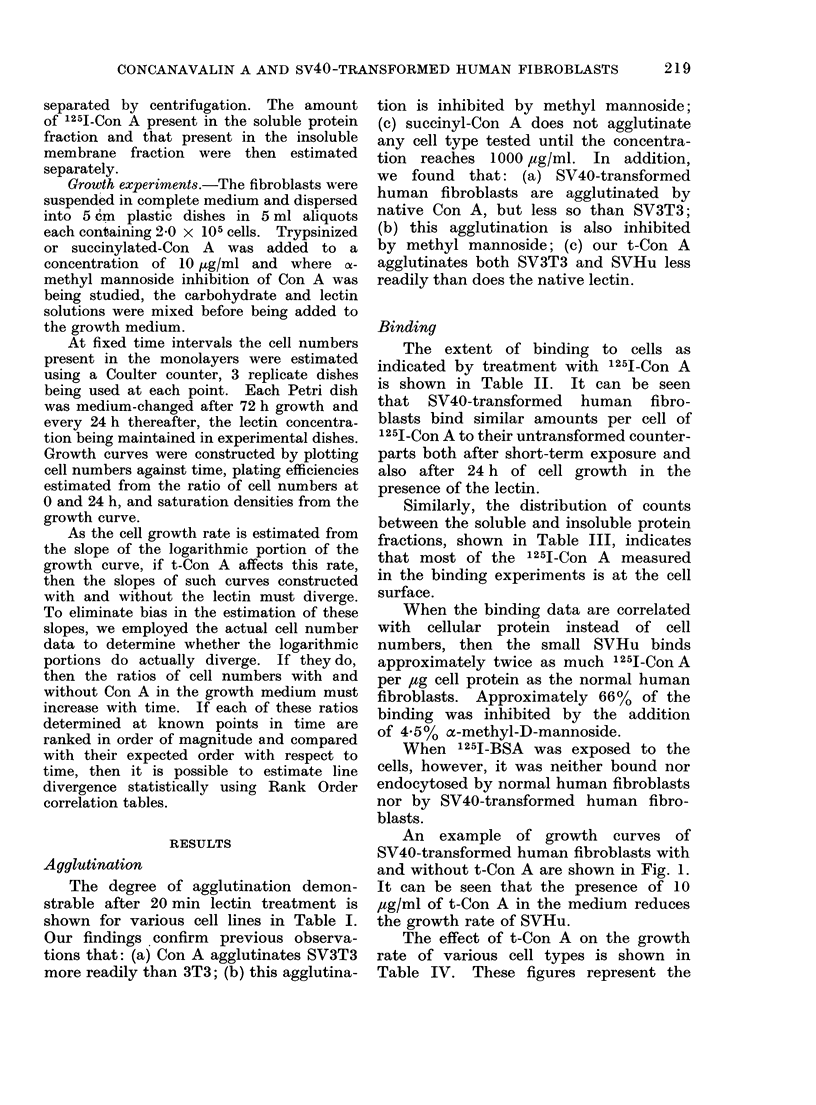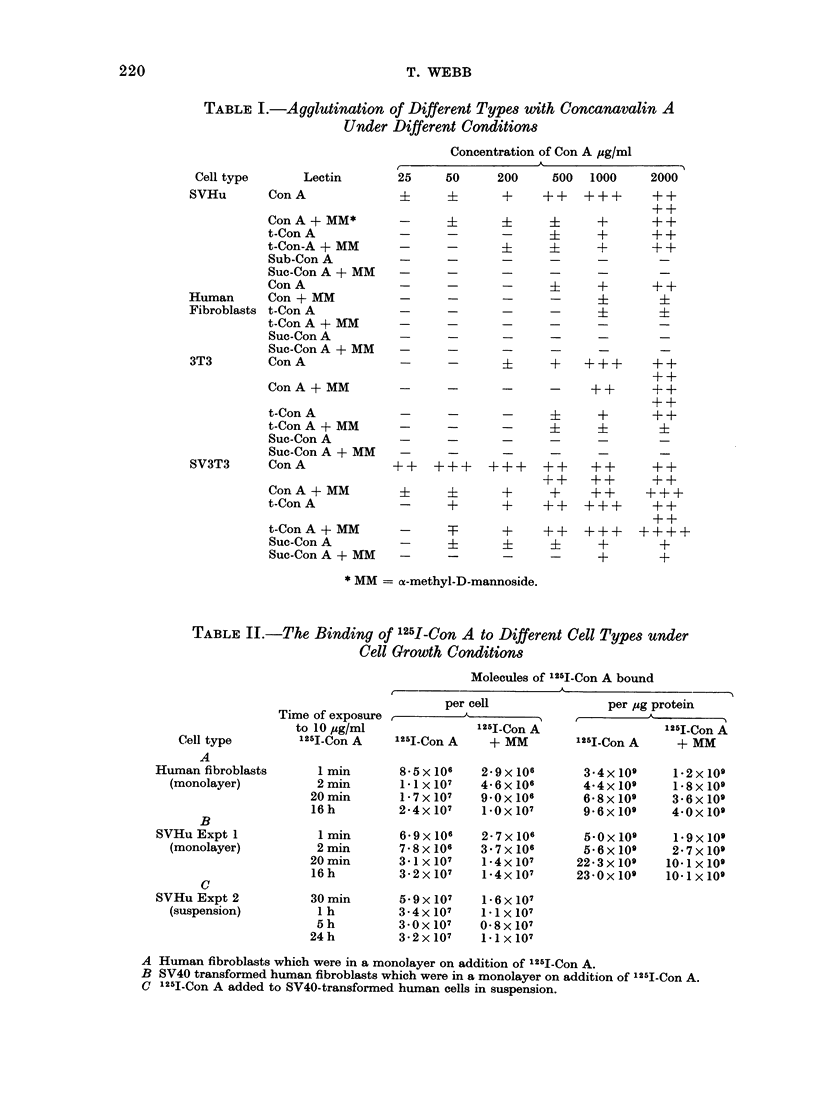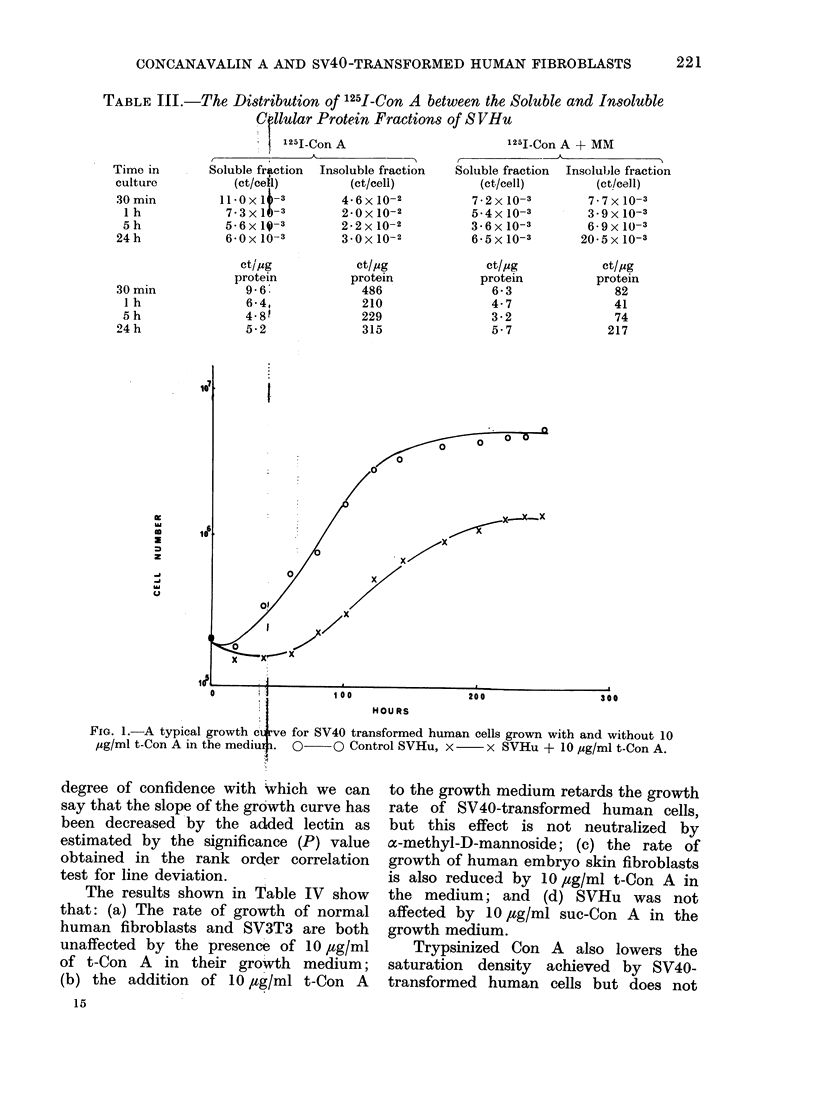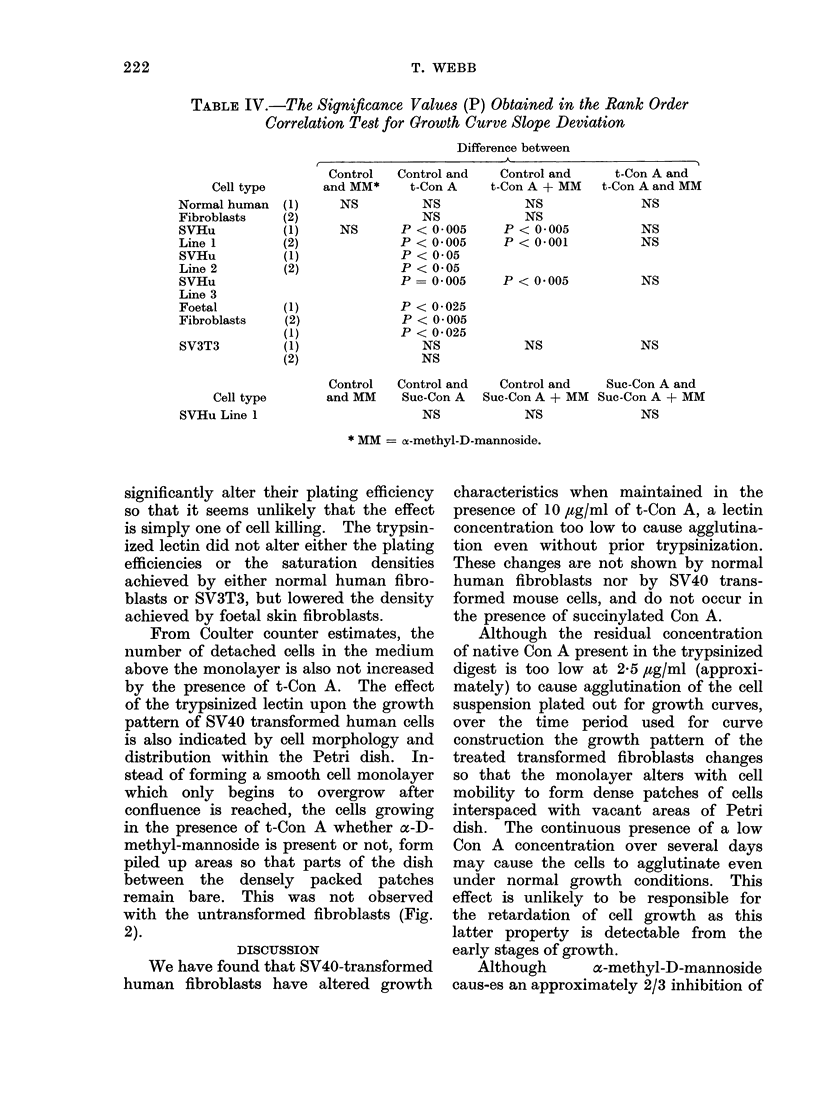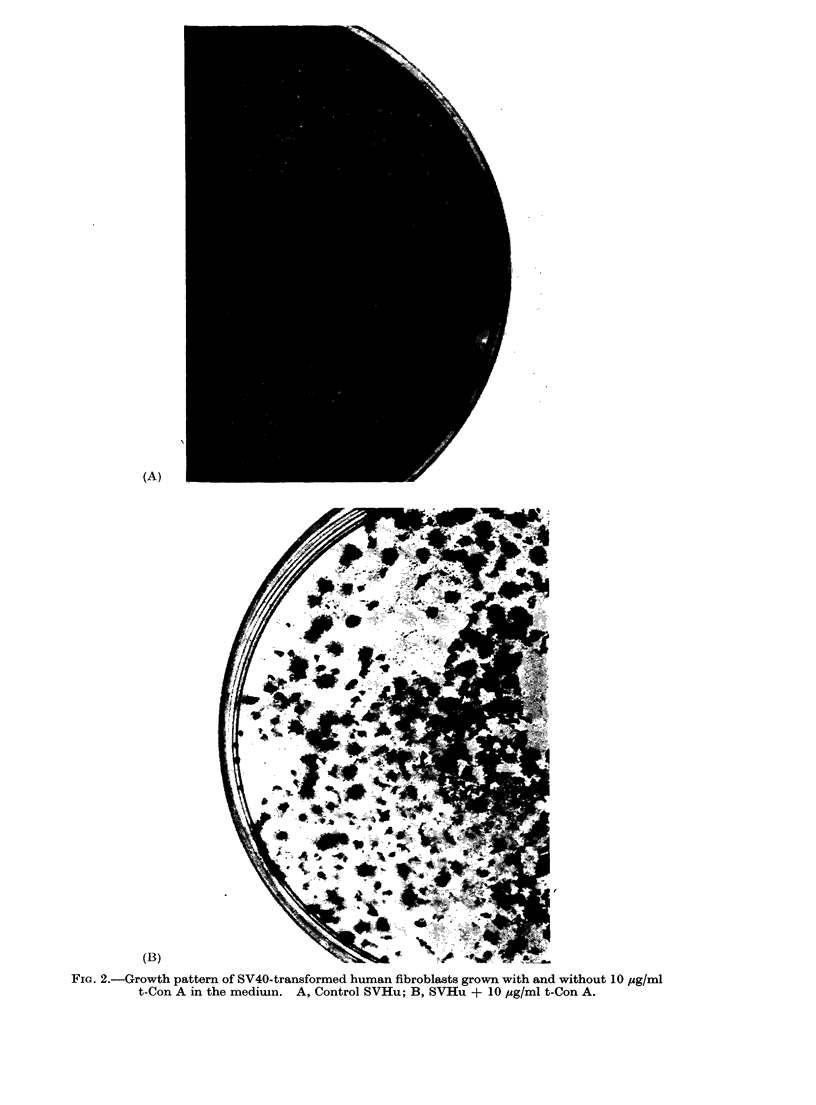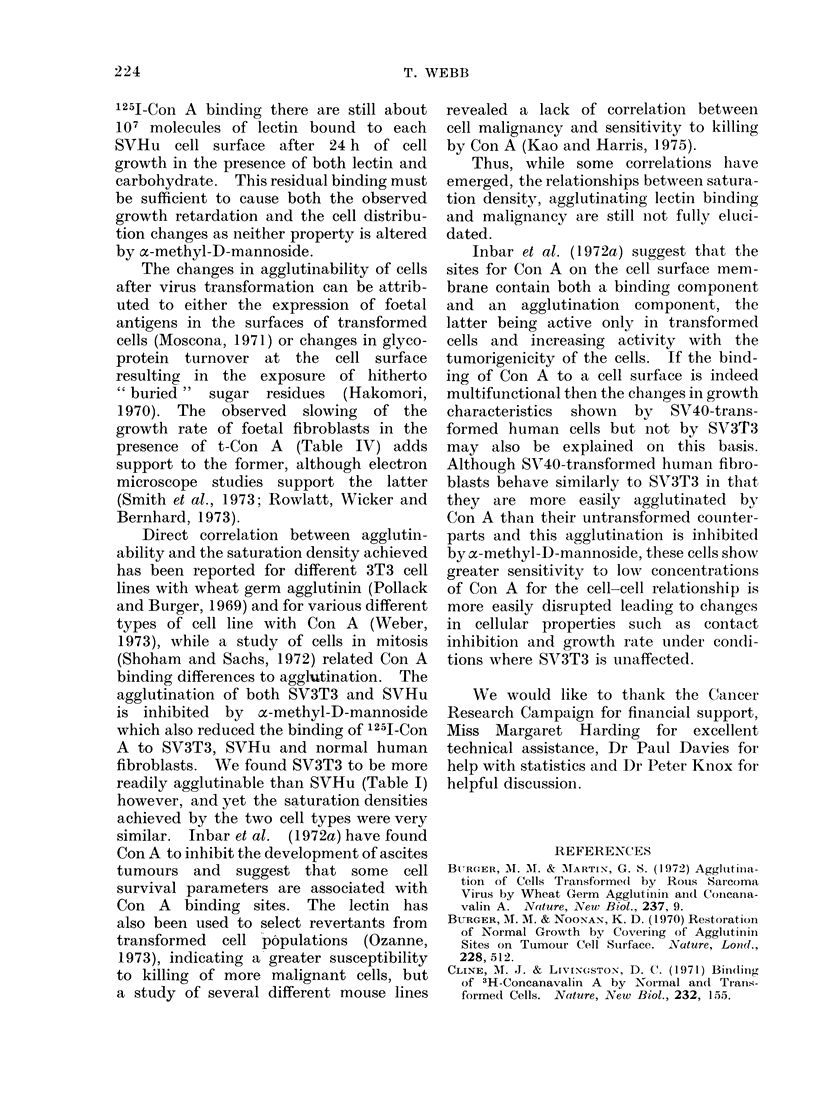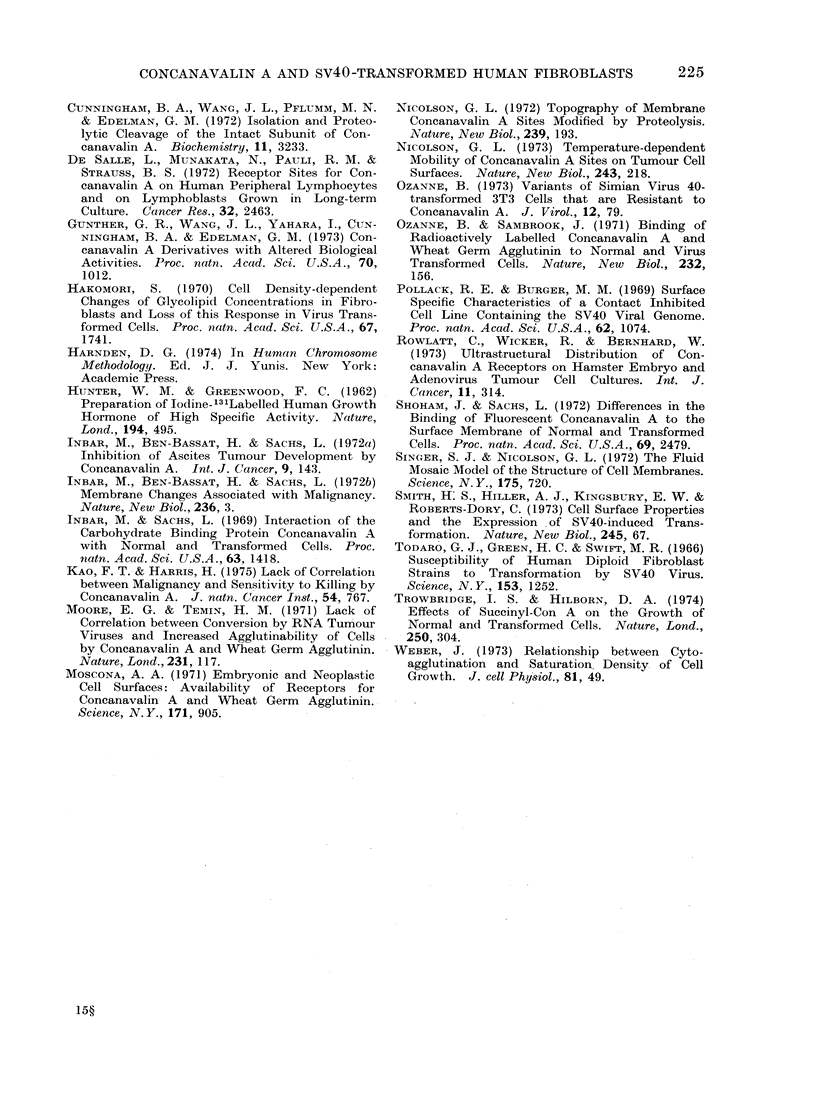# Studies on the relationship between concanavalin A and SV40-transformed human fibroblasts.

**DOI:** 10.1038/bjc.1976.28

**Published:** 1976-02

**Authors:** T. Webb

## Abstract

**Images:**


*Br. J. Cancer* (1976) **33**, 217

### STUDIES ON THE RELATIONSHIP BETWEEN CONCANAVALIN A AND SV40-TRANSFORMED HUMAN FIBROBLASTS

T. WEBB

*From the Department of Cancer Studies, The Medical School, University of Birmingham, Birmingham B15 2TJ*

Received 11 August 1975 Accepted 31 October 1975

**Summary.—The extent of binding of $^{125}$I-Con A to the surface of SV40-transformed human fibroblasts and the degree of agglutination of the cells by the native lectin have been measured. In addition, trypsinized and succinylated Con A have been used to study the effects of the lectin upon certain cell growth parameters. Trypsinized Con A was found to alter the growth rate, the saturation density and the contact inhibition of the transformed cells, an effect not neutralized by α-methyl-D-mannoside.**

THE PLANT lectin Concanavalin A (Con A), which is isolated from Jack bean meal, has been shown to preferentially agglutinate various cell lines which have been transformed by DNA viruses (Inbar, Ben-Bassat and Sachs, 1972*b*). Normal or untransformed cells are agglutinable only after trypsinization (Inbar and Sachs, 1969) and cells transformed by RNA viruses either do not agglutinate (Moore and Temin, 1971) or only do so after enzyme treatment (Burger and Martin, 1972). Normal cells, however, have been shown to bind as much Con A as their transformed counterparts (Cline and Livingston, 1971; Ozanne and Sambrook, 1971), indicating that the receptor sites are present even before transformation but that for agglutination to occur the distribution of sites within the cell membrane must become altered. Singer and Nicolson (1972) suggested a fluid membrane model which could be altered both by transformation and by trypsinization so that the lectin-binding sites could distribute themselves into aggregates. Electron microscopy (Nicolson, 1972) revealed that ferritin–Con A was in fact clustered on trypsinized 3T3 (mouse fibroblast) cells but was distributed randomly on untreated cells. Treatment of cells with 0·1% formalin prior to the addition of Con A did not prevent the clustering of the ferritin label so it was assumed at first that the lectin itself played no part in site aggregation. More recently, however (Nicolson, 1973), SV3T3 (SV40-transformed 3T3) fixed with 2% formaldehyde before treatment with Con A showed a uniform distribution of label, indicating that agglutination site aggregation occurs in transformed cells after lectin treatment and that the cell surface must be capable of fluid movement.

In 1970 Burger and Noonan using Con A rendered monovalent by controlled trypsinization, caused polyoma-transformed 3T3 fibroblasts (Py3T3) to regain the growth characteristics of untransformed 3T3 cells by covering the specific terminal α-D-glucosyl or α-D-mannosyl receptor sites. The lectin, once monovalent, could no longer agglutinate the cells, but by forming an artificial cover layer on the surface caused them to regain contact inhibition of growth.

Further characterization of the monovalent lectin prepared by the method of Burger and Noonan, however, revealed the trypsinized product to be a mixture (Cunningham *et al.*, 1972) so the native lectin tetramer was extensively succinylated to render it unable to agglutinate cells but still able to bind to them. Succinyl-Con A was found not to alter significantly the growth rates of SV3T3, Py3T3 or 3T3 itself (Trowbridge and Hilborn, 1974).

Apart from some studies on human lymphoblasts which may be considered to be E-B virus transformed lymphocytes (De Salle *et al.*, 1972), much of the work utilizing plant agglutinins has been carried out using 3T3 mouse fibroblasts or BHK cells, but not with human material nor with fibroblasts which have been virally transformed before becoming established as a cell line. We have studied the properties of human skin fibroblasts which have been transformed by SV40 within 10 passages of establishing the cells in culture (SVHu) with respect to Con A binding, agglutination and influence upon growth parameters.

### MATERIALS AND METHODS

*Cells.*—Human fibroblasts were established and maintained according to the method of Harnden (1974). Cell maintenance was carried out in Hams F10 with the addition of 10% foetal calf serum (FCS) and antibiotics. Transformed fibroblasts were obtained after infection of cells, between the 5th and 10th passage, with SV40 as reported by Todaro, Green and Swift (1966).

SV3T3 and 3T3 were obtained as a gift from Dr G. D. Clarke of I.C.R.F.

*Concanavalin A.*—The native lectin was bought from Sigma. Trypsinized-Con A (t-Con A) was prepared by the method of Burger and Noonan (1970). After trypsinization was complete, as judged by elimination of SV3T3 cell agglutination, the complexity of the digest was assessed by separation through Sephadex G-75 (Cunningham *et al.*, 1972) using absorbance at 280 nm to visualize the protein present in each fraction. A trace of $^{125}$I-Con A was added as a marker.

Separation of trypsinized Con A by Sephadex G-75 resulted in 2 distinct protein peaks. Estimation of the relative concentrations of these peaks showed that peak 1 contained 3 times as much protein as peak 2 but that peak 2 contained 3 times as much $^{125}$I as peak 1. We could conclude therefore that 25% of the Con A remained in its native state and 75% had become altered, presumably rendered monovalent by trypsinization.

Succinyl-Con A (suc-Con A) was prepared by the method of Gunther *et al.* (1973) and $^{125}$I-Con A by the method of Hunter and Greenwood (1962). As a final step in the production of $^{125}$I-Con A it was co-chromatographed on Sephadex G-75 with the native lectin. $^{125}$I-Bovine serum albumin ($^{125}$I-BSA) was obtained exactly as $^{125}$I-Con A.

α-Methyl-D-mannoside was obtained from 2 sources, Sigma and Cal-Biochem, and was employed at a concentration of 4·5% for inhibition of the lectin.

*Agglutination.*—Cell monolayers were washed 3 times with phosphate buffered saline (PBS), suspended by incubation in 0·04% versene (disodium salt EDTA), rewashed with PBS and resuspended at a concentration of $10^6$ cells/ml. The cell suspensions (0·2 ml) were then added to 0·2 ml of Con A solutions of varying known concentrations contained in the cells of plastic Terasaki plates. After gentle agitation of the plate for 20 min, the degree of cell agglutination was estimated using a $\times$ 10 lens.

*Binding studies.*—The degree to which Con A bound to the cell surface under normal growth conditions was estimated using $^{125}$I-Con A. The labelled lectin was added at a concentration of 10 $\mu$g/ml to the cell growth medium for measured time periods. The cells were then washed 3 times with PBS, suspended with versene, rewashed and finally resuspended in PBS. Aliquots of this suspension were then used for the estimation of cell number by Coulter counter and for measurement of the bound $^{125}$I-Con A.

As the binding measurements were carried out at 37 °C under normal cell growth conditions so that the degree of lectin binding could be correlated with measured growth parameters, it was thought necessary to distinguish between bound lectin and incorporated breakdown products. For this estimation the cells were treated as described except that the suspensions were freeze–thawed 3 times and the membrane fraction separated by centrifugation. The amount of $^{125}$I-Con A present in the soluble protein fraction and that present in the insoluble membrane fraction were then estimated separately.

*Growth experiments*.—The fibroblasts were suspended in complete medium and dispersed into 5 cm plastic dishes in 5 ml aliquots each containing $2 \cdot 0 \times 10^5$ cells. Trypsinized or succinylated-Con A was added to a concentration of 10 $\mu$g/ml and where $\alpha$-methyl mannoside inhibition of Con A was being studied, the carbohydrate and lectin solutions were mixed before being added to the growth medium.

At fixed time intervals the cell numbers present in the monolayers were estimated using a Coulter counter, 3 replicate dishes being used at each point. Each Petri dish was medium-changed after 72 h growth and every 24 h thereafter, the lectin concentration being maintained in experimental dishes. Growth curves were constructed by plotting cell numbers against time, plating efficiencies estimated from the ratio of cell numbers at 0 and 24 h, and saturation densities from the growth curve.

As the cell growth rate is estimated from the slope of the logarithmic portion of the growth curve, if t-Con A affects this rate, then the slopes of such curves constructed with and without the lectin must diverge. To eliminate bias in the estimation of these slopes, we employed the actual cell number data to determine whether the logarithmic portions do actually diverge. If they do, then the ratios of cell numbers with and without Con A in the growth medium must increase with time. If each of these ratios determined at known points in time are ranked in order of magnitude and compared with their expected order with respect to time, then it is possible to estimate line divergence statistically using Rank Order correlation tables.

### RESULTS

### *Agglutination*

The degree of agglutination demonstrable after 20 min lectin treatment is shown for various cell lines in Table I. Our findings confirm previous observations that: (a) Con A agglutinates SV3T3 more readily than 3T3; (b) this agglutination is inhibited by methyl mannoside; (c) succinyl-Con A does not agglutinate any cell type tested until the concentration reaches 1000 $\mu$g/ml. In addition, we found that: (a) SV40-transformed human fibroblasts are agglutinated by native Con A, but less so than SV3T3; (b) this agglutination is also inhibited by methyl mannoside; (c) our t-Con A agglutinates both SV3T3 and SVHu less readily than does the native lectin.

### *Binding*

The extent of binding to cells as indicated by treatment with $^{125}$I-Con A is shown in Table II. It can be seen that SV40-transformed human fibroblasts bind similar amounts per cell of $^{125}$I-Con A to their untransformed counterparts both after short-term exposure and also after 24 h of cell growth in the presence of the lectin.

Similarly, the distribution of counts between the soluble and insoluble protein fractions, shown in Table III, indicates that most of the $^{125}$I-Con A measured in the binding experiments is at the cell surface.

When the binding data are correlated with cellular protein instead of cell numbers, then the small SVHu binds approximately twice as much $^{125}$I-Con A per $\mu$g cell protein as the normal human fibroblasts. Approximately 66% of the binding was inhibited by the addition of 4·5% $\alpha$-methyl-D-mannoside.

When $^{125}$I-BSA was exposed to the cells, however, it was neither bound nor endocytosed by normal human fibroblasts nor by SV40-transformed human fibroblasts.

An example of growth curves of SV40-transformed human fibroblasts with and without t-Con A are shown in Fig. 1. It can be seen that the presence of 10 $\mu$g/ml of t-Con A in the medium reduces the growth rate of SVHu.

The effect of t-Con A on the growth rate of various cell types is shown in Table IV. These figures represent the

TABLE I.—*Agglutination of Different Types with Concanavalin A Under Different Conditions*

| Cell type | Lectin | Concentration of Con A μg/ml 25 | 50 | 200 | 500 | 1000 | 2000 |
|---|---|---|---|---|---|---|---|
| SVHu | Con A | ± | ± | + | ++ | +++ | ++ ++ |
| | Con A + MM* | − | ± | ± | ± | + | ++ |
| | t-Con A | − | − | − | ± | + | ++ |
| | t-Con-A + MM | − | − | ± | ± | + | ++ |
| | Sub-Con A | − | − | − | − | − | − |
| | Suc-Con A + MM | − | − | − | − | − | − |
| | Con A | − | − | − | ± | + | ++ |
| Human | Con + MM | − | − | − | − | ± | ± |
| Fibroblasts | t-Con A | − | − | − | − | ± | ± |
| | t-Con A + MM | − | − | − | − | − | − |
| | Suc-Con A | − | − | − | − | − | − |
| | Suc-Con A + MM | − | − | − | − | − | − |
| 3T3 | Con A | − | − | ± | + | +++ | ++ ++ |
| | Con A + MM | − | − | − | − | ++ | ++ ++ |
| | t-Con A | − | − | − | ± | + | ++ |
| | t-Con A + MM | − | − | − | ± | ± | ± |
| | Suc-Con A | − | − | − | − | − | − |
| | Suc-Con A + MM | − | − | − | − | − | − |
| SV3T3 | Con A | ++ | +++ | +++ | ++ ++ | ++ ++ | ++ ++ |
| | Con A + MM | ± | ± | + | + | ++ | +++ |
| | t-Con A | − | + | + | ++ | +++ | ++ ++ |
| | t-Con A + MM | − | ∓ | + | ++ | +++ | ++++ |
| | Suc-Con A | − | ± | ± | ± | + | + |
| | Suc-Con A + MM | − | − | − | − | + | + |

* MM = α-methyl-D-mannoside.

TABLE II.—*The Binding of* $^{125}$*I-Con A to Different Cell Types under Cell Growth Conditions*

| Cell type | Time of exposure to 10 μg/ml $^{125}$I-Con A | Molecules of $^{125}$I-Con A bound: per cell, $^{125}$I-Con A | per cell, $^{125}$I-Con A + MM | per μg protein, $^{125}$I-Con A | per μg protein, $^{125}$I-Con A + MM |
|---|---|---|---|---|---|
| *A* Human fibroblasts (monolayer) | 1 min | $8 \cdot 5 \times 10^6$ | $2 \cdot 9 \times 10^6$ | $3 \cdot 4 \times 10^9$ | $1 \cdot 2 \times 10^9$ |
| | 2 min | $1 \cdot 1 \times 10^7$ | $4 \cdot 6 \times 10^6$ | $4 \cdot 4 \times 10^9$ | $1 \cdot 8 \times 10^9$ |
| | 20 min | $1 \cdot 7 \times 10^7$ | $9 \cdot 0 \times 10^6$ | $6 \cdot 8 \times 10^9$ | $3 \cdot 6 \times 10^9$ |
| | 16 h | $2 \cdot 4 \times 10^7$ | $1 \cdot 0 \times 10^7$ | $9 \cdot 6 \times 10^9$ | $4 \cdot 0 \times 10^9$ |
| *B* SVHu Expt 1 (monolayer) | 1 min | $6 \cdot 9 \times 10^6$ | $2 \cdot 7 \times 10^6$ | $5 \cdot 0 \times 10^9$ | $1 \cdot 9 \times 10^9$ |
| | 2 min | $7 \cdot 8 \times 10^6$ | $3 \cdot 7 \times 10^6$ | $5 \cdot 6 \times 10^9$ | $2 \cdot 7 \times 10^9$ |
| | 20 min | $3 \cdot 1 \times 10^7$ | $1 \cdot 4 \times 10^7$ | $22 \cdot 3 \times 10^9$ | $10 \cdot 1 \times 10^9$ |
| | 16 h | $3 \cdot 2 \times 10^7$ | $1 \cdot 4 \times 10^7$ | $23 \cdot 0 \times 10^9$ | $10 \cdot 1 \times 10^9$ |
| *C* SVHu Expt 2 (suspension) | 30 min | $5 \cdot 9 \times 10^7$ | $1 \cdot 6 \times 10^7$ | | |
| | 1 h | $3 \cdot 4 \times 10^7$ | $1 \cdot 1 \times 10^7$ | | |
| | 5 h | $3 \cdot 0 \times 10^7$ | $0 \cdot 8 \times 10^7$ | | |
| | 24 h | $3 \cdot 2 \times 10^7$ | $1 \cdot 1 \times 10^7$ | | |

*A* Human fibroblasts which were in a monolayer on addition of $^{125}$I-Con A.
*B* SV40 transformed human fibroblasts which were in a monolayer on addition of $^{125}$I-Con A.
*C* $^{125}$I-Con A added to SV40-transformed human cells in suspension.

TABLE III.—*The Distribution of* $^{125}$*I-Con A between the Soluble and Insoluble Cellular Protein Fractions of SVHu*

| Time in culture | $^{125}$I-Con A: Soluble fraction (ct/cell) | $^{125}$I-Con A: Insoluble fraction (ct/cell) | $^{125}$I-Con A + MM: Soluble fraction (ct/cell) | $^{125}$I-Con A + MM: Insoluble fraction (ct/cell) |
|---|---|---|---|---|
| 30 min | $11 \cdot 0 \times 10^{-3}$ | $4 \cdot 6 \times 10^{-2}$ | $7 \cdot 2 \times 10^{-3}$ | $7 \cdot 7 \times 10^{-3}$ |
| 1 h | $7 \cdot 3 \times 10^{-3}$ | $2 \cdot 0 \times 10^{-2}$ | $5 \cdot 4 \times 10^{-3}$ | $3 \cdot 9 \times 10^{-3}$ |
| 5 h | $5 \cdot 6 \times 10^{-3}$ | $2 \cdot 2 \times 10^{-2}$ | $3 \cdot 6 \times 10^{-3}$ | $6 \cdot 9 \times 10^{-3}$ |
| 24 h | $6 \cdot 0 \times 10^{-3}$ | $3 \cdot 0 \times 10^{-2}$ | $6 \cdot 5 \times 10^{-3}$ | $20 \cdot 5 \times 10^{-3}$ |
| | ct/µg protein | ct/µg protein | ct/µg protein | ct/µg protein |
| 30 min | 9·6 | 486 | 6·3 | 82 |
| 1 h | 6·4 | 210 | 4·7 | 41 |
| 5 h | 4·8 | 229 | 3·2 | 74 |
| 24 h | 5·2 | 315 | 5·7 | 217 |

FIG. 1.—A typical growth curve for SV40 transformed human cells grown with and without 10 µg/ml t-Con A in the medium. O——O Control SVHu, ×——× SVHu + 10 µg/ml t-Con A.

degree of confidence with which we can say that the slope of the growth curve has been decreased by the added lectin as estimated by the significance (*P*) value obtained in the rank order correlation test for line deviation.

The results shown in Table IV show that: (a) The rate of growth of normal human fibroblasts and SV3T3 are both unaffected by the presence of 10 µg/ml of t-Con A in their growth medium; (b) the addition of 10 µg/ml t-Con A to the growth medium retards the growth rate of SV40-transformed human cells, but this effect is not neutralized by α-methyl-D-mannoside; (c) the rate of growth of human embryo skin fibroblasts is also reduced by 10 µg/ml t-Con A in the medium; and (d) SVHu was not affected by 10 µg/ml suc-Con A in the growth medium.

Trypsinized Con A also lowers the saturation density achieved by SV40-transformed human cells but does not

TABLE IV.—*The Significance Values* (P) *Obtained in the Rank Order Correlation Test for Growth Curve Slope Deviation*

| Cell type | | Difference between: Control and MM* | Control and t-Con A | Control and t-Con A + MM | t-Con A and t-Con A and MM |
|---|---|---|---|---|---|
| Normal human | (1) | NS | NS | NS | NS |
| Fibroblasts | (2) | | NS | NS | |
| SVHu | (1) | NS | $P < 0.005$ | $P < 0.005$ | NS |
| Line 1 | (2) | | $P < 0.005$ | $P < 0.001$ | NS |
| SVHu | (1) | | $P < 0.05$ | | |
| Line 2 | (2) | | $P < 0.05$ | | |
| SVHu | | | $P = 0.005$ | $P < 0.005$ | NS |
| Line 3 | | | | | |
| Foetal | (1) | | $P < 0.025$ | | |
| Fibroblasts | (2) | | $P < 0.005$ | | |
| | (1) | | $P < 0.025$ | | |
| SV3T3 | (1) | | NS | NS | NS |
| | (2) | | NS | | |

| Cell type | Control and MM | Control and Suc-Con A | Control and Suc-Con A + MM | Suc-Con A and Suc-Con A + MM |
|---|---|---|---|---|
| SVHu Line 1 | | NS | NS | NS |

* MM = α-methyl-D-mannoside.

significantly alter their plating efficiency so that it seems unlikely that the effect is simply one of cell killing. The trypsinized lectin did not alter either the plating efficiencies or the saturation densities achieved by either normal human fibroblasts or SV3T3, but lowered the density achieved by foetal skin fibroblasts.

From Coulter counter estimates, the number of detached cells in the medium above the monolayer is also not increased by the presence of t-Con A. The effect of the trypsinized lectin upon the growth pattern of SV40 transformed human cells is also indicated by cell morphology and distribution within the Petri dish. Instead of forming a smooth cell monolayer which only begins to overgrow after confluence is reached, the cells growing in the presence of t-Con A whether α-D-methyl-mannoside is present or not, form piled up areas so that parts of the dish between the densely packed patches remain bare. This was not observed with the untransformed fibroblasts (Fig. 2).

### DISCUSSION

We have found that SV40-transformed human fibroblasts have altered growth characteristics when maintained in the presence of 10 μg/ml of t-Con A, a lectin concentration too low to cause agglutination even without prior trypsinization. These changes are not shown by normal human fibroblasts nor by SV40 transformed mouse cells, and do not occur in the presence of succinylated Con A.

Although the residual concentration of native Con A present in the trypsinized digest is too low at 2·5 μg/ml (approximately) to cause agglutination of the cell suspension plated out for growth curves, over the time period used for curve construction the growth pattern of the treated transformed fibroblasts changes so that the monolayer alters with cell mobility to form dense patches of cells interspaced with vacant areas of Petri dish. The continuous presence of a low Con A concentration over several days may cause the cells to agglutinate even under normal growth conditions. This effect is unlikely to be responsible for the retardation of cell growth as this latter property is detectable from the early stages of growth.

Although α-methyl-D-mannoside caus-es an approximately 2/3 inhibition of

FIG. 2.—Growth pattern of SV40-transformed human fibroblasts grown with and without 10 $\mu$g/ml t-Con A in the medium. A, Control SVHu; B, SVHu + 10 $\mu$g/ml t-Con A.

$^{125}$I-Con A binding there are still about $10^7$ molecules of lectin bound to each SVHu cell surface after 24 h of cell growth in the presence of both lectin and carbohydrate. This residual binding must be sufficient to cause both the observed growth retardation and the cell distribution changes as neither property is altered by $\alpha$-methyl-D-mannoside.

The changes in agglutinability of cells after virus transformation can be attributed to either the expression of foetal antigens in the surfaces of transformed cells (Moscona, 1971) or changes in glycoprotein turnover at the cell surface resulting in the exposure of hitherto "buried" sugar residues (Hakomori, 1970). The observed slowing of the growth rate of foetal fibroblasts in the presence of t-Con A (Table IV) adds support to the former, although electron microscope studies support the latter (Smith *et al.*, 1973; Rowlatt, Wicker and Bernhard, 1973).

Direct correlation between agglutinability and the saturation density achieved has been reported for different 3T3 cell lines with wheat germ agglutinin (Pollack and Burger, 1969) and for various different types of cell line with Con A (Weber, 1973), while a study of cells in mitosis (Shoham and Sachs, 1972) related Con A binding differences to agglutination. The agglutination of both SV3T3 and SVHu is inhibited by $\alpha$-methyl-D-mannoside which also reduced the binding of $^{125}$I-Con A to SV3T3, SVHu and normal human fibroblasts. We found SV3T3 to be more readily agglutinable than SVHu (Table I) however, and yet the saturation densities achieved by the two cell types were very similar. Inbar *et al.* (1972*a*) have found Con A to inhibit the development of ascites tumours and suggest that some cell survival parameters are associated with Con A binding sites. The lectin has also been used to select revertants from transformed cell populations (Ozanne, 1973), indicating a greater susceptibility to killing of more malignant cells, but a study of several different mouse lines revealed a lack of correlation between cell malignancy and sensitivity to killing by Con A (Kao and Harris, 1975).

Thus, while some correlations have emerged, the relationships between saturation density, agglutinating lectin binding and malignancy are still not fully elucidated.

Inbar *et al.* (1972*a*) suggest that the sites for Con A on the cell surface membrane contain both a binding component and an agglutination component, the latter being active only in transformed cells and increasing activity with the tumorigenicity of the cells. If the binding of Con A to a cell surface is indeed multifunctional then the changes in growth characteristics shown by SV40-transformed human cells but not by SV3T3 may also be explained on this basis. Although SV40-transformed human fibroblasts behave similarly to SV3T3 in that they are more easily agglutinated by Con A than their untransformed counterparts and this agglutination is inhibited by $\alpha$-methyl-D-mannoside, these cells show greater sensitivity to low concentrations of Con A for the cell–cell relationship is more easily disrupted leading to changes in cellular properties such as contact inhibition and growth rate under conditions where SV3T3 is unaffected.

We would like to thank the Cancer Research Campaign for financial support, Miss Margaret Harding for excellent technical assistance, Dr Paul Davies for help with statistics and Dr Peter Knox for helpful discussion.

### REFERENCES

Burger, M. M. & Martin, G. S. (1972) Agglutination of Cells Transformed by Rous Sarcoma Virus by Wheat Germ Agglutinin and Concanavalin A. *Nature, New Biol.*, **237**, 9.

Burger, M. M. & Noonan, K. D. (1970) Restoration of Normal Growth by Covering of Agglutinin Sites on Tumour Cell Surface. *Nature, Lond.*, **228**, 512.

Cline, M. J. & Livingston, D. C. (1971) Binding of $^3$H-Concanavalin A by Normal and Transformed Cells. *Nature, New Biol.*, **232**, 155.

Cunningham, B. A., Wang, J. L., Pflumm, M. N. & Edelman, G. M. (1972) Isolation and Proteolytic Cleavage of the Intact Subunit of Concanavalin A. *Biochemistry*, **11**, 3233.

De Salle, L., Munakata, N., Pauli, R. M. & Strauss, B. S. (1972) Receptor Sites for Concanavalin A on Human Peripheral Lymphocytes and on Lymphoblasts Grown in Long-term Culture. *Cancer Res.*, **32**, 2463.

Gunther, G. R., Wang, J. L., Yahara, I., Cunningham, B. A. & Edelman, G. M. (1973) Concanavalin A Derivatives with Altered Biological Activities. *Proc. natn. Acad. Sci. U.S.A.*, **70**, 1012.

Hakomori, S. (1970) Cell Density-dependent Changes of Glycolipid Concentrations in Fibroblasts and Loss of this Response in Virus Transformed Cells. *Proc. natn. Acad. Sci. U.S.A.*, **67**, 1741.

Harnden, D. G. (1974) In *Human Chromosome Methodology*. Ed. J. J. Yunis. New York: Academic Press.

Hunter, W. M. & Greenwood, F. C. (1962) Preparation of Iodine-$^{131}$Labelled Human Growth Hormone of High Specific Activity. *Nature, Lond.*, **194**, 495.

Inbar, M., Ben-Bassat, H. & Sachs, L. (1972*a*) Inhibition of Ascites Tumour Development by Concanavalin A. *Int. J. Cancer*, **9**, 143.

Inbar, M., Ben-Bassat, H. & Sachs, L. (1972*b*) Membrane Changes Associated with Malignancy. *Nature, New Biol.*, **236**, 3.

Inbar, M. & Sachs, L. (1969) Interaction of the Carbohydrate Binding Protein Concanavalin A with Normal and Transformed Cells. *Proc. natn. Acad. Sci. U.S.A.*, **63**, 1418.

Kao, F. T. & Harris, H. (1975) Lack of Correlation between Malignancy and Sensitivity to Killing by Concanavalin A. *J. natn. Cancer Inst.*, **54**, 767.

Moore, E. G. & Temin, H. M. (1971) Lack of Correlation between Conversion by RNA Tumour Viruses and Increased Agglutinability of Cells by Concanavalin A and Wheat Germ Agglutinin. *Nature, Lond.*, **231**, 117.

Moscona, A. A. (1971) Embryonic and Neoplastic Cell Surfaces: Availability of Receptors for Concanavalin A and Wheat Germ Agglutinin. *Science, N.Y.*, **171**, 905.

Nicolson, G. L. (1972) Topography of Membrane Concanavalin A Sites Modified by Proteolysis. *Nature, New Biol.*, **239**, 193.

Nicolson, G. L. (1973) Temperature-dependent Mobility of Concanavalin A Sites on Tumour Cell Surfaces. *Nature, New Biol.*, **243**, 218.

Ozanne, B. (1973) Variants of Simian Virus 40-transformed 3T3 Cells that are Resistant to Concanavalin A. *J. Virol.*, **12**, 79.

Ozanne, B. & Sambrook, J. (1971) Binding of Radioactively Labelled Concanavalin A and Wheat Germ Agglutinin to Normal and Virus Transformed Cells. *Nature, New Biol.*, **232**, 156.

Pollack, R. E. & Burger, M. M. (1969) Surface Specific Characteristics of a Contact Inhibited Cell Line Containing the SV40 Viral Genome. *Proc. natn. Acad. Sci. U.S.A.*, **62**, 1074.

Rowlatt, C., Wicker, R. & Bernhard, W. (1973) Ultrastructural Distribution of Concanavalin A Receptors on Hamster Embryo and Adenovirus Tumour Cell Cultures. *Int. J. Cancer*, **11**, 314.

Shoham, J. & Sachs, L. (1972) Differences in the Binding of Fluorescent Concanavalin A to the Surface Membrane of Normal and Transformed Cells. *Proc. natn. Acad. Sci. U.S.A.*, **69**, 2479.

Singer, S. J. & Nicolson, G. L. (1972) The Fluid Mosaic Model of the Structure of Cell Membranes. *Science, N.Y.*, **175**, 720.

Smith, H. S., Hiller, A. J., Kingsbury, E. W. & Roberts-Dory, C. (1973) Cell Surface Properties and the Expression of SV40-induced Transformation. *Nature, New Biol.*, **245**, 67.

Todaro, G. J., Green, H. C. & Swift, M. R. (1966) Susceptibility of Human Diploid Fibroblast Strains to Transformation by SV40 Virus. *Science, N.Y.*, **153**, 1252.

Trowbridge, I. S. & Hilborn, D. A. (1974) Effects of Succinyl-Con A on the Growth of Normal and Transformed Cells. *Nature, Lond.*, **250**, 304.

Weber, J. (1973) Relationship between Cytoagglutination and Saturation Density of Cell Growth. *J. cell Physiol.*, **81**, 49.